# Optogenetic Silencing of Na_v_1.8-Positive Afferents Alleviates Inflammatory and Neuropathic Pain^1^,^2^,^3^

**DOI:** 10.1523/ENEURO.0140-15.2016

**Published:** 2016-03-16

**Authors:** Ihab Daou, Hélène Beaudry, Ariel R. Ase, Jeffrey S. Wieskopf, Alfredo Ribeiro-da-Silva, Jeffrey S. Mogil, Philippe Séguéla

**Affiliations:** 1 Department of Neurology and Neurosurgery, Montreal Neurological Institute, Montreal, Quebec H3A 2B4, Canada; 2 The Alan Edwards Centre for Research on Pain, Montreal, Quebec H3A 0G1, Canada; 3 Department of Pharmacology and Therapeutics, McGill University, Montreal, Quebec H3G 1Y6, Canada; 4 Department of Psychology, McGill University, Montreal, Quebec H3A 1B1, Canada

**Keywords:** allodynia, dorsal root ganglia, inflammation, neuropathy, optogenetics, spinal cord

## Abstract

We report a novel transgenic mouse model in which the terminals of peripheral nociceptors can be silenced optogenetically with high spatiotemporal precision, leading to the alleviation of inflammatory and neuropathic pain. Inhibitory archaerhodopsin-3 (Arch) proton pumps were delivered to Na_v_1.8^+^ primary afferents using the *Na_v_1.8-Cre* driver line. Arch expression covered both peptidergic and nonpeptidergic nociceptors and yellow light stimulation reliably blocked electrically induced action potentials in DRG neurons. Acute transdermal illumination of the hindpaws of *Na_v_1.8-Arch^+^* mice significantly reduced mechanical allodynia under inflammatory conditions, while basal mechanical sensitivity was not affected by the optical stimulation. Arch-driven hyperpolarization of nociceptive terminals was sufficient to prevent channelrhodopsin-2 (ChR2)-mediated mechanical and thermal hypersensitivity in double-transgenic *Na_v_1.8-ChR2^+^-Arch^+^*mice. Furthermore, prolonged optical silencing of peripheral afferents in anesthetized *Na_v_1.8-Arch^+^* mice led to poststimulation analgesia with a significant decrease in mechanical and thermal hypersensitivity under inflammatory and neuropathic conditions. These findings highlight the role of peripheral neuronal inputs in the onset and maintenance of pain hypersensitivity, demonstrate the plasticity of pain pathways even after sensitization has occurred, and support the involvement of Na_v_1.8^+^ afferents in both inflammatory and neuropathic pain. Together, we present a selective analgesic approach in which genetically identified subsets of peripheral sensory fibers can be remotely and optically inhibited with high temporal resolution, overcoming the compensatory limitations of genetic ablations.

## Significance Statement

Selective activation and/or inhibition of peripheral nociceptors allow us to control pain transmission and modulate pain perception. Here, we generated a novel transgenic mouse line in which optical activation of archaerhodopsin-3 (Arch) proton pumps efficiently silenced the activity of Na_v_1.8^+^ nociceptive afferents. Acute and prolonged transdermal illumination of the hindpaws of *Na_v_1.8-Arch^+^* mice reduced mechanical and thermal hypersensitivity under inflammatory and neuropathic conditions, underlining the contribution of the peripheral neuronal component, particularly Na_v_1.8^+^ fibers, in the transmission of evoked pain as well as the development and maintenance of chronic pain. This optogenetic approach can be applied to functionally investigate other subsets of sensory neurons with high temporal precision, and safe genetic delivery of inhibitory opsins may prove useful for clinical applications.

## Introduction

Nociceptors are the primary transducers of noxious and/or potentially damaging stimuli from the periphery to the CNS. Na_v_1.8 is a voltage-gated sodium channel expressed in this subpopulation of primary sensory neurons (Shields et al., 2012). Na_v_1.8 channels play an important role in the generation and propagation of action potentials, thus altering their activity affects neuronal excitability (Garrison et al., 2014; Han et al., 2016). Moreover, Na_v_1.8-expressing neurons have been identified as major players in pain onset and hypersensitivity under chronic conditions. Pharmacological and genetic tools have been used either to interfere with Na_v_1.8 channel functionality (Laird et al., 2002; Gaida et al., 2005; Jarvis et al., 2007; Yu et al., 2011) or to ablate the Na_v_1.8^+^ population in order to assess its role in a variety of pain conditions (Abrahamsen et al., 2008). These studies confirmed the involvement of this neuronal population in pain hypersensitivity under inflammatory conditions, while its role in neuropathic pain has remained controversial (Gaida et al., 2005; Nassar et al., 2005; Villarreal et al., 2005; Joshi et al., 2006; Jarvis et al., 2007; Abrahamsen et al., 2008; Yu et al., 2011).

Pharmacological approaches lack temporal control over drug activity, and target selectivity is a challenge due to the high homology between subtypes of voltage-gated sodium channels. Genetic tools such as knockouts and ablation strategies do not account for compensation at the cellular and circuit levels.

An optogenetic approach might fill these gaps, ensuring a precise spatiotemporal control of the activity of Na_v_1.8-expressing neurons and allowing a peripheral interference with nociceptive transduction. In rats, the expression of archaerhodopsin-3T (ArchT) pumps in the fast-conducting myelinated A-δ high-threshold mechanoreceptors allowed the silencing of these fibers, showing their involvement in withdrawal behaviors under normal and neuropathic (partial sciatic nerve ligation) conditions (Boada et al., 2014). In mice, optical activation of ArchT pumps, expressed under the control of the transient receptor potential vanilloid 1 promoter, resulted in reduced mechanical and thermal sensitivities under normal conditions (Li et al., 2015). However, the latter study did not investigate the analgesic effect of ArchT activation under chronic pain conditions. In another study (Iyer et al., 2014), following viral delivery of halorhodopsin (NpHR) pumps to a subset of small-diameter C-fiber neurons, transdermal yellow light illumination increased sensory thresholds under normal conditions and decreased pain hypersensitivity caused by chronic constriction injury (CCI). Yet, NpHR-mediated analgesia was assessed only in the CCI model, and the neuronal population transduced by the virus was not genetically defined.

Based on our *Na_v_1.8-ChR2^+^* mouse model (Daou et al., 2013) using the *Na_v_1.8-Cre* knock-in construct (Stirling et al., 2005), we specifically delivered inhibitory opsins to Na_v_1.8^+^ neurons to silence the activity of their peripheral terminals with high temporal precision, to assess their involvement in several pain conditions without any ablation, and to validate the analgesic potential of optogenetic actuators. Following the functional assessment of Arch pumps *in vitro*, acute and prolonged transdermal yellow light stimulation of the hindpaws of *Na_v_1.8-Arch^+^* mice were used to evaluate Arch-mediated analgesia under inflammatory and neuropathic conditions. Our results show that acute blockade of Na_v_1.8^+^ terminals reduces pain transmission, and that prolonged inhibition of peripheral input causes short-term analgesia outlasting the optical stimulation. Both strategies support the involvement of Na_v_1.8^+^ afferents in inflammatory and neuropathic pain, and the latter highlights the plasticity of the nociceptive circuit under sensitized conditions. This optogenetic approach provides useful tools to interrogate specific components of the peripheral sensory pathways as well as a promising basis for gene therapy to treat chronic pain.

## Materials and Methods

### Subjects and mouse lines

Five- to sixteen-week-old C57BL/6 mice of both sexes, weighing 20–35 g, were used in this study. All animal procedures were performed in accordance with the McGill University Animal Care Committee regulations. Homozygous *Na_v_1.8-Cre* mice (Stirling et al., 2005) were crossed with homozygous Ai35 mice (The Jackson Laboratory) carrying the *floxed stop-Arch-EGFP* gene in the *ROSA26* locus (Madisen et al., 2012), to generate the *Na_v_1.8-Arch^+^* mouse line. Similarly, mice carrying *Tau-EGFP* in the *ROSA26* locus (from Dr. Ulrich Boehm, University of Hamburg, Hamburg, Germany) were crossed with homozygous *Na_v_1.8-Cre* mice to generate *Na_v_1.8-Tau^+^* control mice. Heterozygous *Na_v_1.8-ChR2^+^* mice were crossed with homozygous Ai35 mice to generate the *Na_v_1.8-ChR2^+^-Arch^+^*double-transgenic mouse line.

### Immunofluorescence

Mice were intracardially perfused with 50 ml of saline (0.9% NaCl), followed by 200 ml of 4% paraformaldehyde (PFA) in 0.01 m PBS, pH 7.4, at room temperature for 30 min. Dorsal root ganglia (DRGs), spinal cord, and glabrous skin were extracted and postfixed in 4% PFA for 24 h at 4°C. Tissue was then cryoprotected in 30% sucrose in PBS overnight at 4°C. To study the spinal cord and glabrous skin, 40-μm-thick sections were cut at −20°C using a cryostat (Leica). All sections were collected as free floating in PBS containing 0.2% Triton X-100 (PBS-T). As for the DRGs, sectioning at 14 μm thickness was performed directly onto gelatin-subbed slides. Sections were initially permeabilized with 50% ethanol for 30 min followed by 1 min of incubation in a 0.3% hydrogen peroxide solution. Sections were washed in PBS-T for 30 min between all incubations. Nonspecific binding of the secondary antibody was blocked by pretreating the sections for 1 h at room temperature in 10% normal goat and donkey serum (Invitrogen) diluted in PBS. The sections were then incubated at 4°C for 24 h with a rabbit anti-calcitonin gene-related protein (CGRP) antibody (Sigma-Aldrich) and a guinea pig anti-purinoceptor P2X_3_ antibody (Neuromics) at a dilution of 1:2000 and 1:25,000, respectively. After several rinses in PBS-T, sections were incubated for 90 min at room temperature with a biotin-conjugated donkey anti-guinea pig IgG (1:200; Jackson ImmunoResearch Laboratories) in PBS, followed by further signal amplification via tyramide (1:75; PerkinElmer) for 7 min. The sections were incubated for 2 h at room temperature with a mixture of streptavidin conjugated to Alexa Fluor 568 (1:200; Molecular Probes) and highly cross-adsorbed goat anti-rabbit IgG conjugated to Alexa Fluor 647 (1:800; Molecular Probes) in 5% normal goat and donkey serum in PBS-T. Finally, the sections were washed, mounted on gelatin-subbed slides (spinal cord and glabrous skin), air dried, and coverslipped with antifading mounting medium (Aqua PolyMount, Polysciences). Slides were stored at 4°C until examination under a Zeiss LSM 710 confocal microscope.

### Cell culture and DRG preparation

DRGs were extracted from adult *Na_v_1.8-Arch^+^* mice or adult *Na_v_1.8-ChR2^+^-Arch^+^* mice and kept in sterile ice-cold 1× HBSS (Invitrogen) throughout the dissection. DRGs were then incubated in 5 ml of HBSS containing 1.4 mg/ml dispase (Sigma-Aldrich) and 1.1 mg/ml collagenase type II (Sigma-Aldrich) for 45 min at 37°C. Following the enzymatic reaction, DRGs were washed twice with 10 ml of culture media [F-12 media (Invitrogen), containing 10% FBS, 1% l-glutamine, 1% penicillin, and 1% streptomycin] and then mechanically triturated using fire-polished Pasteur pipettes. The dissociated neurons were finally plated onto five 35 mm culture dishes (2 ml/dish; Sarstedt) previously coated with laminin (BD Biosciences) and poly-d-lysine (Sigma-Aldrich). Cells were incubated for 24 h at 37°C and 5% CO_2_ prior to electrophysiological recording.

### Whole-cell electrophysiology

Whole-cell patch-clamp recordings on DRG neurons were conducted at room temperature, 24 h after plating. The internal solution (pH 7.2) of the pipette contained the following (in mm): 130 K-gluconate, 1 MgCl_2_, 10 HEPES, 5 EGTA, 3 MgATP, and 0.4 GTP. The bath solution, pH 7.4, contained the following (in mm): 152 NaCl, 5 KCl, 2 CaCl_2_, 1 MgCl_2_, 10 HEPES, and 10 glucose. Patch pipettes had a tip resistance of 5–10 MΩ. Electrophysiological recordings were conducted using an Axopatch 200B amplifier, digitized with a Digidata 1322A interface (Axon Instruments). Traces were acquired and analyzed using pClamp 8.2 software (Axon Instruments). Recordings were low-pass filtered at 2 and 5 KHz in voltage- and current-clamp configurations, respectively. Multimode optic fibers (200 μm diameter; Thorlabs), coupled to diode-pumped solid-state lasers of specific wavelengths (473 nm blue laser, Laserglow Technologies; or 589 nm yellow laser, Dragon Lasers), were used for optical stimulation of DRG neurons. Stimulation parameters are specified in each condition. Light intensities were measured using a PM100A power meter coupled to a S130C photodiode sensor (Thorlabs) and analyzed using LabVIEW 8.5 software.

### Behavioral experiments under normal and inflammatory conditions

Due to the nature of the experiments, particularly the use of visible light, trials were not conducted blindly. Mice were habituated for 1 h prior to testing. Thermal sensitivity was measured using the radiant heat paw-withdrawal test. A blue filter (True Blue #196, Lee Filters) was placed on the light source to filter out the orange-yellow component and prevent nonspecific activation of Arch pumps. Mechanical sensitivity was assessed using an automated von Frey fiber (dynamic plantar aesthesiometer, Ugo Basile) applied to the mid-plantar hindpaw. The nontreated hindpaw was used as a control.

The analgesic effect of acute optical stimulation was assessed under normal and inflammatory conditions. Yellow light was applied to the plantar surface of the hindpaw simultaneously with the mechanical or thermal stimulus. The light was administered continuously using a 1 mm optic fiber (Prizmatix) coupled to a 589 nm laser.

Two inflammatory conditions were used to assess the effect of yellow light stimulation on mechanical hypersensitivity in *Na_v_1.8-Arch^+^* mice, as follows: an intradermal injection of 10 μl of capsaicin (0.05%; Sigma-Aldrich) into the right hindpaw, leading to mechanical allodynia lasting at least 60 min postinjection, and an intraplantar injection of 20 μl of zymosan (1 mg/ml; Sigma-Aldrich) into the left hindpaw, leading to mechanical allodynia lasting up to 24 h postinjection. Control *Na_v_1.8-Arch^+^* mice were injected with inflammatory compounds, but no optical stimulation was applied while assessing mechanical sensitivity.

To test the effect of prolonged silencing of Na_v_1.8^+^ fibers on inflammatory pain, mechanical and thermal sensitivities were assessed before and after a prolonged (1 h) yellow light stimulation of the injected hindpaw. The hour-long stimulations were conducted under 2% isoflurane anesthesia with a 3 s ON, 1 s OFF pulsing frequency. The light beam (0.3–0.45 mW/mm^2^) covered the entire plantar surface. Control *Na_v_1.8-Arch^+^* mice were injected with the inflammatory compound and placed under isoflurane for 1 h without any optical stimulation. Mechanical and thermal measurements were taken at least 1 h poststimulation to give the mice enough time to recover from anesthesia. An intraplantar injection of 20 μl zymosan (1 mg/ml) was used to investigate the mechanical modality, while an intraplantar injection of 25 μl of emulsified complete Freund’s adjuvant (CFA; 0.5 mg/ml; EMD Millipore) was used to study the thermal modality, because CFA produced more consistent and reproducible thermal hypersensitivity than zymosan. Zymosan-induced mechanical hypersensitivity was measured at 2, 4, 6, 8, and 24 h postinjection, while CFA-mediated thermal hypersensitivity was assessed at 2, 24, 48, and 72 h postinjection.

### Behavioral testing on the double-opsin mouse line

To determine whether yellow light can prevent blue light-induced ChR2-mediated hypersensitivity, the left hindpaw of *Na_v_1.8-ChR2^+^-Arch^+^*mice was stimulated either with blue light alone (“Ipsi-Blue”) or with blue and yellow light simultaneously (“Ipsi-Blue + Yellow”) (blue, 2 Hz at 2.3 mW/mm^2^; yellow, 3 s ON, 1 s OFF at 0.3–0.45 mW/mm^2^; 30 min for mechanical, 1 h for thermal). Thermal and mechanical sensitivities were measured before, and 1, 3, and 24 h post-stimulation. All stimulations were performed under isoflurane anesthesia, and right hindpaws were used as controls.

### Spared nerve injury and mechanical allodynia

Six-week-old mice were deeply anesthetized using 2% isoflurane. The left anterior thigh of the animal was shaved and disinfected using 70% isopropyl alcohol. To induce the spared nerve injury (SNI) neuropathy, an incision was made through the anterior surface of the left thigh to expose the sciatic nerve at the trifurcation level. The common peroneal and tibial nerves were tightly ligated with 6.0 silk and sectioned distal to the ligation. The sural nerve was left intact. Sham surgery involved exposing the nerve without damaging it. The skin was closed using interrupted sutures (6.0 silk). A 1 ml subcutaneous saline injection (0.9%) was administered to replenish fluids, and the mouse recovered on a heating pad until it was ambulatory and was then returned to the cage. An eye ointment (Tears Naturale PM) was applied to keep the eyes lubricated during the procedure.

Mechanical sensitivity of both hindpaws was measured up to 9 weeks postsurgery, using von Frey monofilaments (Stoelting). Monofilaments allow the precise targeting of the lateral part of the hindpaw. At 3, 4, 6, and 9 weeks postsurgery, the neuropathic paw was exposed to a prolonged (1 h, 3 s ON, 1 s OFF, 0.3-0.45 mW/mm^2^) yellow light stimulation under 2% isoflurane anesthesia (“SNI-Light”). Mechanical sensitivity was measured up to 24 h poststimulation. To control for the isoflurane effect, neuropathic *Na_v_1.8-Arch^+^* mice were placed under isoflurane for 1 h without optical stimulation (“SNI-Iso”). To control for any nonspecific yellow light effect, sham *Na_v_1.8-Arch^+^* mice were submitted to the same optical stimulation as the neuropathic mice, and their mechanical sensitivity was followed over a similar time course poststimulation.

### Statistical analysis

All data are represented as the mean ± SEM. All statistical tests were performed in GraphPad Prism version 6, and statistical significance was set at *p* < 0.05.

For testing under normal conditions, measurements on both hindpaws were combined and averaged across animals for each intensity/stimulation paradigm. Comparison was performed with the baseline measurements using the paired Student’s *t* test (Table 1).

**Table 1:** Paired Student’s *t* test

Data				Test	*p*
a	Figure 2A	Force (g)	No light vs 0.25 mW/mm^2^	Paired Student’s *t* test	0.0707

For experiments with the double-opsin mice and all the inflammatory tests (capsaicin, zymosan, CFA), data were averaged for each hindpaw at each time point across animals, and comparison was performed between the “Ipsi-Light” (ipsilateral hindpaws stimulated optically) and “Ipsi-Control” (ipsilateral hindpaws not stimulated) conditions (for inflammatory tests) and between “Ipsi-Blue” and “Ipsi-Blue + Yellow” (for double-opsin experiments) at each time point, using repeated-measures two-way ANOVA followed by the Sidak *post hoc* test (Tables 2, 3).

Regarding the SNI experiments, data were averaged for each hindpaw at each time point across animals, and comparisons were performed between the “SNI-Light” and “SNI-Iso” conditions, using repeated-measures two-way ANOVA followed by the Sidak *post hoc* test (Tables 2, 3), and between each post-light (“Post”) time point and before light (“Pre”) in the SNI-Light group, using repeated-measures one-way ANOVA followed by the Dunnett’s *post hoc* test (Tables 4, 5).

## Results

### Distribution and functionality of Arch-EGFP in sensory pathways

**Figure 1.**
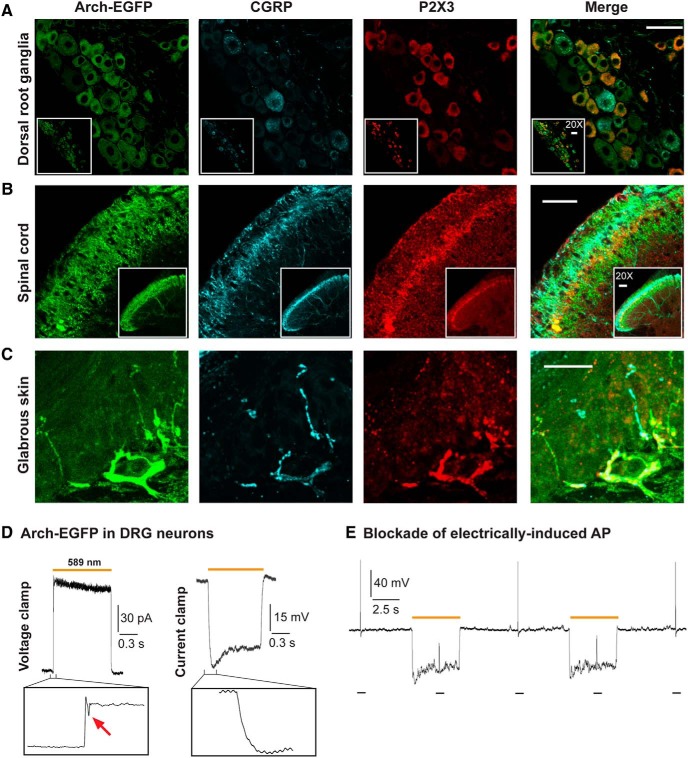
Distribution and functionality of Arch-EGFP pumps in the peripheral sensory pathways of *Na_v_1.8-Arch^+^* mice. Confocal micrographs showing the fluorescence of Arch-EGFP (green), CGRP immunostaining (blue), P2X_3_ labeling (red), and the merge. ***A***, Arch-EGFP colocalizes with either P2X_3_ or CGRP in dorsal root ganglia neurons, validating its selective expression in nociceptors. ***B***, Arch-EGFP fluorescence overlaps with CGRP and P2X_3_ labeling in laminae I and II of the dorsal horn of spinal cord. ***C***, Arch-EGFP colocalizes with CGRP and P2X_3_ in free nerve endings in the lower and upper dermis of glabrous skin. Insets are at lower magnification (20×). Scale bars, 50 μm. ***D***, Representative yellow (589 nm) light-induced outward photocurrent and membrane hyperpolarization in a DRG neuron. Magnification shows an inward deflection in the voltage-clamp trace (arrow), illustrating a proton-mediated ASIC-like current. This inward current did not translate into a depolarization in the current-clamp trace. ***E***, Arch-mediated blockade of electrically induced action potentials (10 ms, 0.4 nA current injection) in DRG neurons. Optical stimulation was continuous (intensity, 0.83 mW/mm^2^). V_h_ = −60 mV in voltage-clamp configuration; and the resting membrane potential was −62.23 ± 2.92 mV in current-clamp configuration (*n* = 8–9 cells).

To assess the expression profile of transgenic Arch-EGFP pumps in the peripheral pathways of *Na_v_1.8-Arch^+^* mice, we determined their colocalization with the peptidergic and nonpeptidergic markers CGRP and P2X_3_, respectively. In DRG neurons, almost all P2X_3_- and CGRP-immunopositive cells were EGFP-positive, with the P2X_3_ and CGRP signals being mutually exclusive (Fig. 1*A*). EGFP fluorescence was further detected in superficial laminae of the dorsal horn of the spinal cord, indicating the efficient trafficking of Arch pumps to their central targets. It colocalized with CGRP labeling in laminae I and II_o_, and P2X_3_ labeling in lamina II_i_, and extended ventrally to lamina III, which is innervated by myelinated fibers (Fig. 1*B*). Transgenic opsins were effectively transported to the periphery, where EGFP fluorescence overlapped with P2X_3_ and CGRP labeling in the deep and superficial layers of the glabrous skin bordering the dermal–epidermal junction (Fig. 1*C*).

The functionality of Arch-EGFP was then tested in cultured DRG neurons. Typical outward photocurrents (2.04 ± 0.3 pA/pF) were recorded under yellow light (589 nm) stimulation, leading to significant and reproducible membrane hyperpolarizations (−24.15 ± 3.51 mV; Fig. 1*D*). Light-evoked hyperpolarizations were sufficient to block electrically induced action potentials in DRG neurons (Fig. 1*E*), showing the ability of Arch pumps to inhibit Na_v_1.8^+^ nociceptor activity *in vitro*. Taking a closer look at Arch-mediated photocurrents, we detected small inward currents at the beginning of light application (Fig. 1*D*, left). However, these acid-sensing ion channel (ASIC)-like currents (Zeng et al., 2015) did not translate into depolarization, as shown in the current-clamp trace (Fig. 1*D*, right). Also, both the size and duration of the depolarizing currents were significantly smaller than those of the Arch-mediated photocurrents and hyperpolarizations. Altogether, although a decrease in extracellular pH can be depolarizing, the overall effect of optical stimulations is inhibitory.

### Analgesic effects of acute optical stimulation *in vivo*

**Figure 2.**
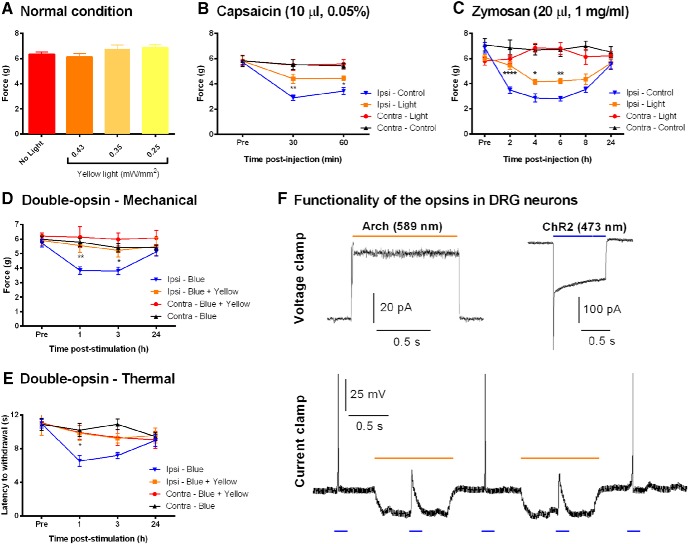
Analgesic effect of acute yellow light stimulation under normal and inflammatory conditions in *Na_v_1.8-Arch^+^* mice, and under prolonged blue light stimulation in *Na_v_1.8-ChR2^+^-Arch^+^*mice. ***A***, Mechanical sensitivity of *Na_v_1.8-Arch^+^* mice under normal conditions, using different yellow light (589 nm) intensities (*n* = 8–17 mice/condition; paired Student’s *t* test, No light vs 0.25 mW/mm^2^ measurements, *p* = 0.0707^a^; Table 1). ***B***, ***C***, Optical stimulation reduced mechanical allodynia under capsaicin- and zymosan- induced inflammation. Yellow light (0.25 mW/mm^2^) was applied to the hindpaw of *Na_v_1.8-Arch^+^* mice simultaneously with the mechanical stimulus at each time point postinjection (*n* = 6–13 mice/condition). ***D***, ***E***, Simultaneous application of yellow and blue light to the hindpaw of *Na_v_1.8-ChR2^+^-Arch^+^*mice prevented the development of blue light-induced mechanical (***D***) and thermal (***E***) hypersensitivity. Post-treatment measurements were taken without any optical stimulation (*n* = 6–13 mice/condition). Symbols represent the mean ± SEM of hindpaw withdrawal thresholds and latencies before (Pre) and after (Post) treatment. Data were analyzed using repeated-measures two-way ANOVA followed by Sidak *post hoc* test. Significance (*) is reported between Ipsi-Light and Ipsi-Control (***B***, ***C***) and between Ipsi-Blue and Ipsi-Blue + Yellow (***D***, ***E***). *p < 0.05, **p < 0.01, ****p < 0.0001. ***F***, Representative yellow (589 nm) and blue (473 nm) light-induced photocurrents in DRG neurons from *Na_v_1.8-ChR2^+^-Arch^+^*mice. Arch-mediated membrane hyperpolarization was sufficient to block ChR2-induced action potentials (50 ms pulses) in the same neuron (*n* = 4–5 cells). Light intensity was 0.78 mW/mm^2^ (blue) and 0.83 mW/mm^2^ (yellow).

We next tested whether acute optical silencing of Na_v_1.8^+^ afferents interferes with sensory perception in behaving *Na_v_1.8-Arch^+^* mice. Mechanical sensitivity was assessed under normal and inflammatory conditions. Transdermal yellow light illumination of the hindpaw was applied simultaneously with the mechanical stimulus. We found that mechanical thresholds were not altered under normal conditions, using a range of light intensities (0.25-0.43 mW/mm^2^; Fig. 2*A*; *n* = 8–17 mice/condition; paired Student’s *t* test used to compare “No light” vs “0.25 mW/mm^2^” measurements, *p* = 0.0707^a^; Table 1). However, optical stimulation (0.25 mW/mm^2^) significantly reduced capsaicin- and zymosan-induced mechanical allodynia [Fig. 2*B*,*C*; for capsaicin, *n* = 6 mice/condition, comparison between Ipsi-Light and Ipsi-Control was performed using repeated-measures two-way ANOVA^b^ (Table 2); followed by the Sidak multiple-comparisons test at 30 min, *p* = 0.0018^k^; and at 60 min, *p* = 0.0424^k^ (Table 3); for zymosan, *n* = 12–13 mice/condition, comparison between Ipsi-Light and Ipsi-Control was performed using repeated-measures two-way ANOVA^c^ (Table 2); followed by the Sidak multiple-comparisons test at 2 h, *p* < 0.0001^l^; at 4 h, *p* = 0.0104^l^; and at 6 h, *p* = 0.0036^l^ (Table 3)], indicating effective Arch-mediated analgesia under inflammatory conditions. These findings are consistent with the role of Na_v_1.8^+^ neurons in mediating inflammatory pain but not in setting basal mechanical sensitivity under normal conditions (Abrahamsen et al., 2008).

**Table 2:** Repeated-measures two-way ANOVA

Data			Source of variation											
			Light			Time			Interaction			Matching		
			dfn, dfd	*F*	*p*	dfn, dfd	*F*	*p*	dfn, dfd	*F*	*p*	dfn, dfd	*F*	*p*
b	Figure 2B	Force (g)	1, 10	9.206	0.0126	2, 20	49.6	<0.0001	2, 20	4.775	0.0202	10, 20	2.371	0.0481
c	Figure 2C	Force (g)	1, 23	12.63	0.0017	5, 115	44.5	<0.0001	5, 115	7.556	<0.0001	23, 115	2.298	0.0021
d	Figure 2D	Force (g)	1, 16	8.039	0.0119	3, 48	7.704	0.0003	3, 48	3.194	0.0317	16, 48	2.353	0.0115
e	Figure 2E	Latency (s)	1, 17	6.192	0.0235	3, 51	6.753	0.0006	3, 51	2.111	0.1103	17, 51	1.418	0.1672
f	Figure 3A, left	Force (g)	1, 12	11.97	0.0047	5, 60	26.74	<0.0001	5, 60	2.285	0.0574	12, 60	2.566	0.0082
g	Figure 3A, middle	Force (g)	1, 13	5.769	0.032	5, 65	32.44	<0.0001	5, 65	4.424	0.0016	13, 65	1.016	0.4471
h	Figure 3A, right	Force (g)	1, 16	0.2823	0.6025	5, 80	68.17	<0.0001	5, 80	4.293	0.0016	16, 80	1.734	0.0567
i	Figure 3B, right	Latency (s)	1, 12	0.5795	0.4612	8, 96	63.15	<0.0001	8, 96	2.216	0.0327	12, 96	1.634	0.0947
j	Figure 4, left	50% threshold (g)	1, 19	8.91	0.0076	5, 95	1.826	0.1151	5, 95	1.861	0.1085	19, 95	3.687	<0.0001

dfn, df numerator; dfd, df denominator.

**Table 3:** Multiple comparisons following repeated-measures two-way ANOVA

Data			Test	*p*
k	Figure 2B	At 30 min: Ipsi-Light vs Ipsi-Control	Sidak	0.0018
		At 60 min: Ipsi-Light vs Ipsi-Control	Sidak	0.0424
l	Figure 2C	At 2 h: Ipsi-Light vs Ipsi-Control	Sidak	<0.0001
		At 4 h: Ipsi-Light vs Ipsi-Control	Sidak	0.0104
		At 6 h: Ipsi-Light vs Ipsi-Control	Sidak	0.0036
m	Figure 2D	At 1 h: Ipsi-Blue vs Ipsi-Blue + Yellow	Sidak	0.0034
		At 3 h: Ipsi-Blue vs Ipsi-Blue + Yellow	Sidak	0.0215
n	Figure 2E	At 1 h: Ipsi-Blue vs Ipsi-Blue + Yellow	Sidak	0.0106
o	Figure 3A, left	At 2 h: Ipsi-Light vs Ipsi-Control	Sidak	0.0141
		At 4 h: Ipsi-Light vs Ipsi-Control	Sidak	0.0128
p	Figure 3A, middle	At 4 h: Ipsi-Light vs Ipsi-Control	Sidak	<0.0001
q	Figure 3A, right	At 6 h: Ipsi-Light vs Ipsi-Control	Sidak	0.0007
r	Figure 3B, right	At 24 (+1) h: Ipsi-Light vs Ipsi-Control	Sidak	0.0331
s	Figure 4, left	At 1 h: SNI-Iso vs SNI-Light	Sidak	0.0221
		At 2 h: SNI-Iso vs SNI-Light	Sidak	0.0375

The effect of acute yellow light stimulation on the thermal modality was also investigated. Unfortunately, simultaneous application of laser light with the light from the Hargreaves device increased thermal sensitivity in an intensity-dependent manner (data not shown). This was also the case on control *Na_v_1.8-Tau^+^* mice, showing that this effect is Arch-independent (data not shown). This is most likely due to additive heating, making this approach inappropriate to assess Arch-mediated analgesia for the thermal modality.

The analgesic effect observed under inflammation was significant but partial. This can be due to insufficient Arch-driven hyperpolarizations of peripheral afferents to completely block nociceptive transmission. Alternatively, the absence of Arch expression in subpopulations of sensitized terminals may explain the partial reversal of mechanical allodynia. For instance, the presence of Arch-negative nociceptors would allow the transduction and transmission of nociceptive inputs from the periphery despite the optical stimulation. Furthermore, low-threshold Aβ mechanoreceptors play an essential role in mediating mechanical allodynia under inflammatory conditions. Thus, the absence of Arch pumps in these fibers enables them to normally transmit mechanical stimuli to the CNS, accounting, at least partially, for the pain hypersensitivity. Another factor that could explain this partial analgesia is a limited penetration of light into the skin, leading to incomplete inhibition of deeper primary afferent terminals. To address these possibilities, we generated the double-opsin *Na_v_1.8-ChR2^+^-Arch^+^*mice, in which ChR2 channels and Arch pumps are coexpressed in the same Na_v_1.8^+^ neurons, overcoming the limitation of having Arch-negative sensitized terminals. Also, the use of blue light to induce and yellow light to prevent sensitization ensures the targeting of the same afferents since both stimuli have the same physical nature. We have shown that prolonged blue light stimulation of the hindpaw of anesthetized *Na_v_1.8-ChR2^+^* mice induces mechanical as well as thermal hypersensitivity lasting up to 24 h poststimulation (Daou et al., 2013). Using either blue light alone or blue and yellow light together on *Na_v_1.8-ChR2^+^-Arch^+^* mice, we assessed the ability of Arch pumps to prevent ChR2-mediated hypersensitivity. When used alone, blue light induced a significant reduction in withdrawal thresholds and latencies lasting up to 24 h; however, this sensitization was completely prevented in the presence of yellow light in both mechanical and thermal modalities [Fig. 2*D*,*E*; for mechanical, *n* = 6–12 mice/condition: comparison between Ipsi-Blue and Ipsi-Blue + Yellow was performed using repeated-measures two-way ANOVA^d^ (Table 2); followed by the Sidak multiple-comparisons test, at 1 h, *p* = 0.0034^m^; and at 3 h, *p* = 0.0215^m^ (Table 3); for thermal, *n* = 6–13 mice/condition: comparison between Ipsi-Light and Ipsi-Control was performed using repeated-measures two-way ANOVA^e^ (Table 2); followed by the Sidak multiple-comparisons test, at 1 h, *p* = 0.0106^n^ (Table 3)]. These results are consistent with our *in vitro* recordings in cultured DRG neurons from *Na_v_1.8-ChR2^+^-Arch^+^* mice, in which Arch-mediated hyperpolarizations suppressed ChR2-induced action potentials in the same neurons (Fig. 2*F*). These findings support the effectiveness of Arch pumps in silencing peripheral afferents and preventing the onset of sensitization when expressed with high penetrance in the target neurons. Also, the *Na_v_1.8-ChR2^+^-Arch^+^* mouse line provides a transgenic model in which Na_v_1.8^+^ neurons can be bidirectionally controlled with high spatiotemporal precision.

### Prolonged silencing of Na_v_1.8^+^ afferents leads to poststimulation analgesia

We further tested the effect of a prolonged inhibition of peripheral sensory pathways on inflammatory and neuropathic pain hypersensitivity. Anesthetized *Na_v_1.8-Arch^+^* mice were subjected to hour-long yellow light stimulations at different time points after the induction of inflammation or nerve injury. Mechanical thresholds and thermal latencies were measured at least 1 h after light exposure. This approach allowed us to overcome the complications resulting from the simultaneous application of yellow light and thermal stimulus, and to assess the analgesic effects of prolonged peripheral silencing.

**Figure 3.**
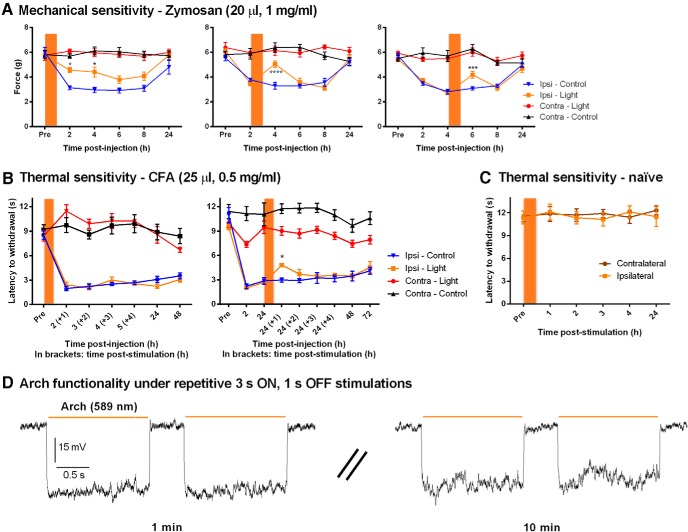
Analgesic effect of prolonged yellow light stimulation under inflammatory conditions in *Na_v_1.8-Arch^+^* mice. Orange bars represent the hour-long yellow light stimulations. ***A***, Prolonged silencing of Na_v_1.8^+^ fibers led to poststimulation analgesia, reducing zymosan-mediated mechanical allodynia. Optical stimulation was performed right after, 2 h after, and 4 h after zymosan injection (*n* = 7–11 mice/condition). ***B***, Prolonged yellow light stimulation reduced CFA-induced thermal hypersensitivity when applied 24 h after CFA injection but not right after (*n* = 5–7 mice/condition). ***C***, Hour-long stimulation does not affect thermal sensitivity in naive *Na_v_1.8-Arch^+^* mice (*n* = 6 mice). ***D***, Representative traces showing consistent and reproducible Arch-mediated hyperpolarizations in cultured DRG neurons at 1 and 10 min during a sustained 3 s ON, 1 s OFF stimulation protocol (*n* = 3 cells). Symbols represent the mean ± SEM of hindpaw withdrawal thresholds and latencies before (Pre) and after (Post) treatment. Significance between Ipsi-Light and Ipsi-Control measured using repeated-measures two-way ANOVA followed by Sidak *post hoc* test: *p < 0.05, ***p < 0.001, ****p < 0.0001.

Under zymosan-induced inflammation, optical stimulation delayed the onset of mechanical allodynia when applied right after injection, while it caused a significant and transient reduction of allodynia when applied 2 and 4 h after zymosan injection (Fig. 3*A*, left, *n* = 7 mice/condition; middle, *n* = 7–8 mice/condition; right, *n* = 7–11 mice/condition). In all panels, comparison between Ipsi-Light and Ipsi-Control was performed using repeated-measures two-way ANOVA^f,g,h^ (Table 2) followed by the Sidak multiple-comparisons test (Fig. 3*A*, left: at 2 h, *p* = 0.0141^°^; and at 4 h, *p* = 0.0128^°^; middle, at 4 h, *p* = < 0.0001^p^; right, at 6 h, *p* = 0.0007^q^; Table 3). In the latter cases, the analgesia peaked at 1 h and returned to control levels 3 h after optical stimulation.

Arch-mediated analgesia was milder when evaluating thermal hypersensitivity under CFA-induced inflammation. No analgesia was detected when light was applied right after CFA injection, yet, a transitory increase in thermal latencies was measured when light was applied 24 h postinjection [Fig. 3*B*, right; *n* = 7 mice/condition; comparison between Ipsi-Light and Ipsi-Control was performed using repeated-measures two-way ANOVA^i^ (Table 2); followed by the Sidak multiple-comparisons test, at 24 (+1) h, *p* = 0.0331^r^ (Table 3)]. No redness, edema, or sign of neurogenic inflammation was detected following hour-long stimulations of the hindpaw. Furthermore, prolonged exposure to yellow light did not alter thermal sensitivity of naive *Na_v_1.8-Arch^+^* mice (Fig. 3*C*). Also, the effect of yellow light under hyperalgesic conditions (CFA) was controlled for using the control transgenic *Na_v_1.8-Tau^+^* mouse line, where CFA-injected *Na_v_1.8-Tau^+^* mice were exposed to hour-long light exposure 24 h postinjection. No effects on CFA-mediated thermal hypersensitivity were detected in these mice (data not shown), indicating that the transitory effect observed in *Na_v_1.8-Arch^+^* mice (Fig. 3*B*, right) is Arch-mediated.

The partial reversal in both mechanical and thermal modalities can be attributed to (1) the possibility that Arch-negative afferents play an important role in the onset and maintenance of pain hypersensitivity, (2) the incomplete blockade of peripheral inputs due to limited light penetration or potency of the stimulus, and/or (3) central sensitization mechanisms independent of peripheral inputs.

To investigate whether Arch pumps desensitize during prolonged optical stimulations, we conducted *in vitro* recordings on cultured DRG neurons from *Na_v_1.8-Arch^+^* mice, using the same stimulation protocol (3 s ON, 1 s OFF) as that used *in vivo*. Ten-minute-long recordings under sustained optical stimulation showed that Arch-mediated hyperpolarizations are consistent and replicable over time, indicating that (1) prolonged illumination does not alter the activity and recovery of Arch pumps (Fig. 3*D*), and (2) neuronal excitability and input resistance remained unchanged. This further suggests that the analgesic effects observed in behavioral experiments are not due to the decreased excitability of peripheral afferents but rather to central plasticity where the blockade of peripheral inputs to the spinal cord reduces central sensitization, leading to decreased pain hypersensitivity.

**Figure 4.**
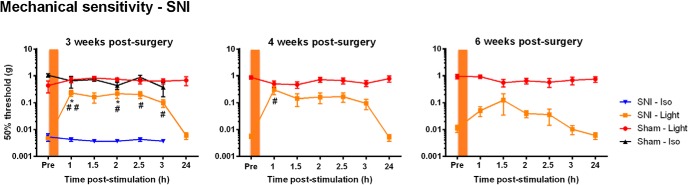
Analgesic effect of prolonged optical stimulation under neuropathic conditions in *Na_v_1.8-Arch^+^* mice. Orange bars represent the hour-long optical stimulations. Prolonged yellow light application on the neuropathic hindpaw decreased SNI-induced mechanical allodynia, showing partial analgesia lasting up to 24 h poststimulation, at 3, 4, and 6 weeks after SNI surgery (*n* = 7–14 mice for SNI; *n* = 3–7 mice for sham). Symbols represent the mean ± SEM of hindpaw withdrawal thresholds before (Pre) and after (Post) treatment. Significance between SNI-Light and SNI-Iso, measured using repeated-measures two-way ANOVA followed by Sidak *post hoc* test: *p < 0.05. Significance between each Post time point and Pre in the SNI-Light group, measured using repeated-measures one-way ANOVA followed by Dunnett’s *post hoc* test: #*p* < 0.05, ##*p* < 0.01.

Using the SNI model to produce a neuropathic condition, we tested whether prolonged inhibition of Na_v_1.8^+^ fibers can reduce mechanical allodynia. Hour-long illuminations of the hindpaw markedly decreased mechanical hypersensitivity at 3, 4, and 6 weeks after SNI surgery, with a transient effect lasting up to 24 h poststimulation [Fig. 4, *n* = 7–14 mice for SNI, *n* = 3–7 mice for Sham; Fig. 4, left, comparison between SNI-Iso and SNI-Light was performed using repeated-measures two-way ANOVA^j^ (Table 2); followed by the Sidak multiple-comparisons test, at 1 h, *p* = 0.0221^s^; at 2 h, *p* = 0.0375^s^ (Table 3); comparison between each Post time point and Pre in the SNI-Light group was conducted using repeated-measures one-way ANOVA^t^ (Table 4); followed by the Dunnett’s multiple-comparisons test, at 1 h Post, *p* = 0.0093^v^; at 2 h Post, *p* = 0.0321^v^; at 2.5 h Post, *p* = 0.0181^v^; at 3 h Post, *p* = 0.0483^v^ (Table 5). Fig. 4, middle, comparison between each Post time point and Pre in the SNI-Light group was performed using repeated-measures one-way ANOVA^u^ (Table 4); followed by the Dunnett’s multiple-comparisons test, at 1 h Post, *p* = 0.0437^w^ (Table 5)]. Analgesia was most pronounced at 3 weeks and gradually decreased at 4 and 6 weeks after SNI (Fig. 4). Arch-mediated analgesia was completely lost when optical stimulation was applied 9 weeks after SNI (data not shown), indicating that the peripheral inhibition of Na_v_1.8^+^ nociceptors becomes less efficient in reducing mechanical pain at later stages of neuropathy. By showing that silencing the activity of their peripheral terminals alleviates mechanical allodynia, especially at the early stages of neuropathy, these results clearly demonstrate the involvement of Na_v_1.8^+^ nociceptors in SNI-induced neuropathic pain.

**Table 4:** Repeated-measures one-way ANOVA

Data			Time			Matching		
			dfn, dfd	*F*	*p*	dfn, dfd	*F*	*p*
t	Figure 4, left	50% threshold (g)	3.54, 46.02	3.783	0.0123	13, 65	3.687	0.0002
u	Figure 4, middle	50% threshold (g)	2.142, 27.85	3.304	0.0486	13, 52	3.288	0.0011

dfn, df numerator; dfd, df denominator.

**Table 5.** Multiple comparisons following repeated-measures one-way ANOVA

Data			Test	*p*
v	Figure 4, left	SNI-Light: Pre vs 1 h Post	Dunnett’s	0.0093
		SNI-Light: Pre vs 2 h Post	Dunnett’s	0.0321
		SNI-Light: Pre vs 2.5 h Post	Dunnett’s	0.0181
		SNI-Light: Pre vs 3 h Post	Dunnett’s	0.0483
w	Figure 4, middle	SNI-Light: Pre vs 1 h Post	Dunnett’s	0.0437

## Discussion

Taking advantage of the *Na_v_1.8-Cre* mouse line, we delivered the inhibitory pump Arch to Na_v_1.8^+^ afferents, generating a novel transgenic model in which interference with peripheral nociceptive transmission can be optogenetically achieved with high spatiotemporal precision. This silencing strategy allowed us to specifically manipulate the Na_v_1.8^+^ neuronal subpopulation while overcoming the compensatory effects encountered using other genetic strategies such as knockdowns, knockouts, or cell ablation.

The Na_v_1.8^+^ population covers peptidergic and nonpeptidergic nociceptors as well as a subset of low-threshold mechanoreceptors. Under inflammatory and neuropathic conditions, central sensitization and plasticity as well as disinhibition in the spinal cord occur; input from low-threshold Aβ fibers can now be relayed to nociceptive circuits in the dorsal horn, leading to pain perception. This phenomenon accounts, at least partially, for the mechanical allodynia in chronic pain conditions. Thus, optogenetic silencing of Na_v_1.8^+^ myelinated A-fibers would further reduce pain hypersensitivity, which is in accordance with the analgesic effects we observe.

Acute yellow light illumination of the hindpaws of *Na_v_1.8-Arch^+^*mice did not alter their mechanical thresholds under normal conditions. These results fit with those showing that von Frey thresholds were unaffected when the whole Na_v_1.8^+^ subpopulation of neurons was eliminated (Abrahamsen et al., 2008), indicating that these neurons are not essential in setting basal mechanical sensitivity. The same transdermal optical stimulation reduced mechanical allodynia induced by capsaicin and zymosan, demonstrating that a brief and acute silencing of Na_v_1.8^+^ fibers is sufficient to alleviate inflammatory pain. Furthermore, a prolonged inhibition of Na_v_1.8^+^ afferents delayed the onset and/or transiently reduced inflammatory mechanical allodynia. This shows that Arch-mediated inhibition of the terminals of peripheral nociceptors is effective in decreasing inflammatory pain, confirming the involvement of the Na_v_1.8^+^ subpopulation in mediating hypersensitivity under inflammatory conditions. The mechanisms of inhibition in the two stimulation protocols may be different: under acute light stimulation, analgesia may be mediated by direct hyperpolarization of the sensory fibers transducing the mechanical stimulus since both stimuli are applied simultaneously; following long illumination, synaptic changes in the spinal circuitry may occur due to peripheral silencing of nociceptive inputs, leading to decreased central sensitization and reduced allodynia. Hour-long yellow light stimulations of CFA-injected *Na_v_1.8-Arch^+^*mice led to a transitory increase in thermal latencies when applied 24 h postinjection, but not right after injection. These findings indicate that Arch pumps are not capable of blocking or delaying the onset of CFA-induced thermal hyperalgesia. This apparent discrepancy with the zymosan results might be explained by the difference in the sensory modality investigated or by the strength of the CFA stimulation that could not be reversed as effectively.

Under neuropathic conditions, the role of Na_v_1.8^+^ neurons remained unclear (Nassar et al., 2005; Joshi et al., 2006; Jarvis et al., 2007; Abrahamsen et al., 2008). Using the SNI model, we showed that prolonged hyperpolarization of Na_v_1.8^+^ afferents produces a significant and transient alleviation of mechanical allodynia at 3 and 4 weeks after surgery, proving the contribution of the Na_v_1.8^+^ population and demonstrating the analgesic effectiveness of our optogenetic strategy in neuropathic pain. Analgesia may be explained by Arch-mediated blockade of the spontaneous hyperactivity of peripheral nociceptors, presumably leading to a modified sensitivity of their terminals and/or altered spinal connectivity affecting central sensitization. Interestingly, the analgesic effect of yellow light decreased over time after surgery, suggesting that peripheral inputs in general, and specifically inputs from the Na_v_1.8^+^ population, are not essential for the maintenance of mechanical allodynia.

Arch pumps extrude protons from the cell. This can lead to changes in intracellular and/or extracellular pH affecting neuronal activity. Previous reports have shown that changes in intracellular pH (pH_i_) following Arch activation in cultured neurons do not exceed 0.1–0.15 U. Alkalinization of the intracellular space is very limited, most likely due to intrinsic stabilization mechanisms in the neuron (e.g., transporters, exchangers), preventing the development of proton gradients and a further increase in pH_i_ (Chow et al., 2010, 2012). Furthermore, during *in vivo* recordings in mice and macaques, spike waveform and frequency were not affected before and after prolonged (several minutes) Arch-mediated inhibition (Chow et al., 2012). Extracellularly, proton efflux can lead to a transient decrease in pH and therefore the activation of proton-gated channels. A recent study (Zeng et al., 2015) showed that Arch activation induces ASIC currents in HEK293 cells and cortical neurons overexpressing ASIC1a. This was detected as an inward deflection in voltage-clamp recordings *in vitro* under green light illumination. These inward currents were also detected in our DRG recordings under yellow light stimulation, yet their amplitudes were smaller and did not cause any significant depolarization. This difference in current size is normal since the levels of expression of endogenous acid-sensitive channels are lower than those observed in overexpression systems. Thus, although the decrease in extracellular pH can be sensitizing for peripheral terminals, the overall effect of the optical stimulations is analgesic, indicating that the inhibitory component of Arch activation overcomes any sensitizing or excitatory effect of transient acid-evoked currents.

In conclusion, the *Na_v_1.8-Arch^+^*mouse line represents a constitutive model in which silencing of peripheral nociceptors is achieved remotely with high spatiotemporal precision. We can predict that such an optogenetic approach will be implemented using next-generation inhibitory opsins such as ChloC (Wietek et al., 2014) or the newly identified *Gt*ACR anion channels (Govorunova et al., 2015), possibly leading to more pronounced analgesia. Moreover, considering the large heterogeneity of sensory neurons revealed by recent molecular profiling studies (Goswami et al., 2014; Flegel et al., 2015; Usoskin et al., 2015), our silencing strategy will be beneficial for selective targeting and functional interrogation of other subsets of sensory neurons, including non-nociceptive populations. Selective delivery of inhibitory opsins to genetically identified neurons will allow the control of pain circuits *in vivo*, setting the stage for a potential use in humans for the treatment of intractable chronic pain.

## Synthesis

The decision was a result of the Reviewing Editor Maiken Nedergaard and the peer reviewers coming together and discussing their recommendations until a consensus was reached. A fact-based synthesis statement explaining their decision and outlining what is needed to prepare a revision is listed below. The following reviewer(s) agreed to reveal their identity: Ru-Rong Ji

Review #1:

The authors have appropriately addressed my concerns. This study will provide research community a useful optogenetic tool to study the unique role of primary sensory neurons. Although the authors have tried their best, an hour long stimulation of acid channel could produce some unwanted effects.
